# Supplementary figures and images for: Body Composition as a Predictor of the Survival in Anal Cancer
Source: Cancers (Basel). 2022 Sep 18;14(18):4521. doi: 10.3390/cancers14184521 (PMC9496941; doi:10.3390/cancers14184521)

Suppl. Figures

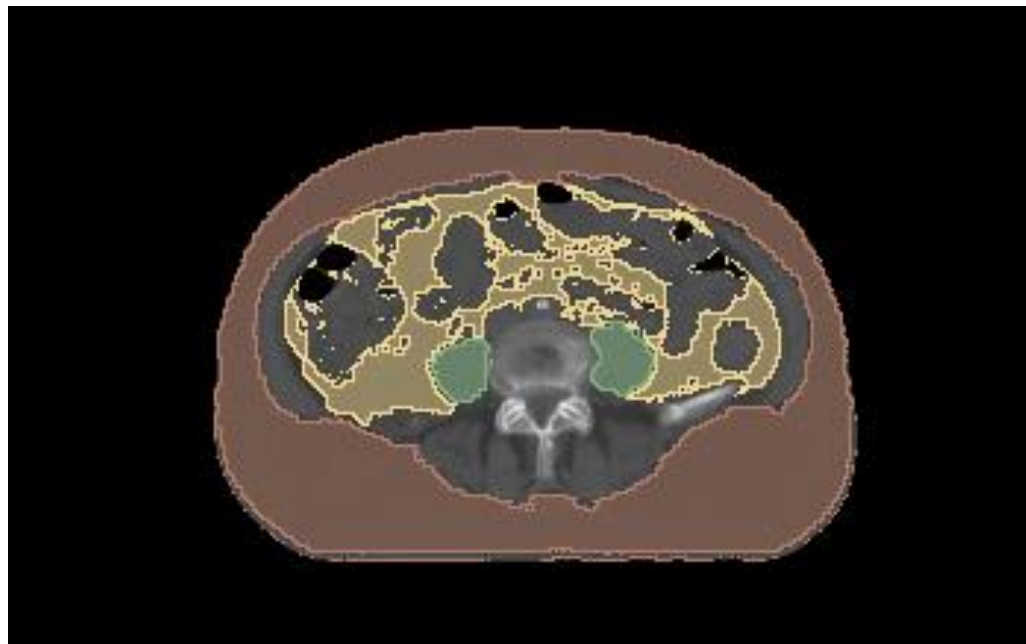

Suppl. Figure S1: representative CT slide at the level of L4-5.

Supplement: Supplementary file 1 [file cancers-14-04521-s001.zip › cancers-1874722-supplementary.pdf]
